# Feminization of Longnose Dace (*Rhinichthys cataractae*) in the Oldman River, Alberta, (Canada) Provides Evidence of Widespread Endocrine Disruption in an Agricultural Basin

**DOI:** 10.6064/2012/521931

**Published:** 2012-08-02

**Authors:** Joyce S. Evans, Leland J. Jackson, Hamid R. Habibi, Michael G. Ikonomou

**Affiliations:** ^1^Department of Biological Sciences, University of Calgary, 2500 University Drive, NW, Calgary, AB, Canada T2N 1N4; ^2^TERA Environmental Consultants, Suite 1100, 815-8th Avenue SW, Calgary, AB, Canada T2P 3P2; ^3^Institute of Environmental Toxicology, University of Calgary, 2500 University Drive, NW, Calgary, AB, Canada T2N 1N4; ^4^Institute of Ocean Sciences, Fisheries and Oceans Canada, Sidney, BC, Canada V8L 1Z4

## Abstract

We sampled an abundant, native minnow (Longnose dace—*Rhinichthys cataractae*) throughout the Oldman River, Alberta, to determine physiological responses and possible population level consequences from exposure to compounds with hormone-like activity. Sex ratios varied between sites, were female-biased, and ranged from just over 50% to almost 90%. Histological examination of gonads revealed that at the sites with >60% females in the adult population, there was up to 38% occurrence of intersex gonads in fish identified through visual examination of the gonads as male. In the majority of intersex gonad cases, there was a large proportion (approx., 50%) of oocytes within the testicular tissue. In male dace, vitellogenin mRNA expression generally increased with distance downstream. We analyzed river water for 28 endocrine disrupting compounds from eight functional classes, most with confirmed estrogen-like activity, including synthetic estrogens and hormone therapy drugs characteristic of municipal wastewater effluent, plus natural hormones and veterinary pharmaceuticals characteristic of livestock production. The spatial correlation between detected chemical residues and effects to dace physiology indicate that multiple land uses have a cumulative impact on dace in the Oldman River and effects range from altered gene regulation to severely female-biased sex ratios.

## 1. Background

Environmental contaminants with hormone-like activity, also known as endocrine disrupting compounds (EDCs), can adversely impact normal physiological processes by acting as agonists (mimicking hormones), antagonists (blocking receptors), and by interfering with hormone synthesis/metabolism [1]. Many natural and synthetic hormones elicit estrogen-like activity; for example, natural estrogens and compounds with a similar structure can bind to estrogen receptors and cause an estrogen-mediated response [2]. 

Wastewater treatment plant effluent is a common source of natural hormones and estrogen-like compounds in aquatic environments [3]. Growing populations globally discharge waste to aquatic systems and in some areas more than 50% of river flow is wastewater effluent [1]. Wastewater treatment plants have not historically been designed to remove EDCs from process water and discharge EDCs at very low, yet biologically active concentrations [4] presumably due, in part, to synergistic interactions [5] of contaminants in complex mixtures. In addition to natural hormones, a number of pharmaceuticals such as ethinyl estradiol, a potent estrogen present in many oral contraceptives, is occasionally detected downstream of urban centers in wastewater treatment plant effluent [6]. Additionally, xenoestrogens such as nonylphenol and bisphenol A have unintended estrogen-like properties [1]. 

Runoff from intensive agricultural landscapes may also add EDCs to aquatic environments [7]. Significant concentrations of natural estrogen and testosterone have been found in runoff from cattle holding facilities [8] and fields fertilized with chicken litter (a mixture of bird excreta, feathers, waste feed, and bedding material) [9]. In the Oldman River, Alberta, *α*-zearalanol, a nonsteroidal growth promoter used in cattle, has been measured in river water near high feedlot densities [10]. Furthermore, compounds with androgen-like activity are known to exist in aquatic environments [1]. In addition to testosterone, *β*-Trenbolone, a potent synthetic androgen, has been detected in cattle feedlot effluent in areas inhabited by fathead minnows (*Pimephales promelas*) [7]. Female fathead minnows from contaminated sites were found to be masculinized and displayed increased testosterone production and decreased estrogen production. Female mosquito fish (*Gambusia* sp.) living in a stream exposed to pulp and paper mill effluents have been found to be strongly masculinized [11]. Thus, estrogenic and androgenic compounds may be part of complex mixtures in the aquatic environment, complicating a mechanistic understanding of EDCs and their effects on fish. 

Endocrine disruption in fish has been well studied and research has shown that estrogen-mimicking compounds alter biological processes that range from gene regulation to morphological development [1]. Male fish exposed to estrogen-like compounds produce vitellogenin, an egg yolk precursor protein that is present in higher concentrations in the females of oviparous organisms [12]. Elevated levels of vitellogenin in male fish, which is naturally not produced, indicates exposure to estrogen or estrogen-mimicking contaminants [13]. Estrogen receptor (ER), which mediates the effects of estrogens, is influenced by estrogens and estrogen-mimicking compounds [14, 15]. In laboratory exposures goldfish significantly increased gene expression for the three estrogen receptor subtypes (ER*α*, ER*β*1, and ER*β*2) after treatment with estradiol [15]. In field populations, exposure to compounds with estrogen-like activity may lead to intersex testes [16]. Fish with severely intersex gonads have been found in English rivers in the settlement lagoons of wastewater treatment plants [1]. White suckers (*Catostomus commersoni*) with intersex testes and female-biased sex ratios were found downstream of wastewater treatment plants in Colorado [17, 18]. The severity of intersex tissue can range from having a few oocytes within a predominately testicular tissue matrix [19] to gonads that appear to be normal ovaries yet the fish's genotype is presumed to be male [20]. The extensiveness of intersex likely depends on the degree of exposure to estrogen-like compounds [1]. 

 Exposure to estrogenic-like compounds is not the only form of endocrine disruption shown to occur in aquatic organisms. Statistical modeling of the concentrations and activities of different types of EDCs in English rivers and the feminization of wild male fish show that antiandrogenic compounds also have a feminizing effect [21]. EDCs may mimic or antagonize progesterones, which act as pheromones and are released into the surroundings by one sex during spawning to facilitate a response in the other [1]. Synthetic progesterones have been successfully used to attract Chinese black sleepers (*Bostrichthys sinensis*) and induce spawning in experimental pools [22]. Compounds that act in this manner have the potential to affect spawning and, therefore, long-term viability of natural populations in aquatic environments. 

 Here we build on earlier studies [10, 23] conducted in 2005 and 2006 to assess the temporal consistency of their findings, to confirm skewed sex ratios by means of gonad histology and to determine the frequency and extent of intersex gonads. Evidence of widespread endocrine disruption was shown in three Alberta rivers within the South Saskatchewan River Basin in longnose dace (*Rhinichthys cataractae*), an abundant minnow species [10]. Previously, Jeffries et al. [10, 23] detected synthetic estrogen in river water, female-biased sex ratios, and elevated levels of vitellogenin in male dace. Jeffries et al. [10, 23] assigned sex by visual gonad examination only and no histological analysis was conducted. In the absence of a known appropriate sex-determinant gene marker in longnose dace, determination of sex ratios by histological techniques would be more reliable than visual identification alone and provides an opportunity to investigate the incidence of intersex testes. We note that the significant female bias observed at some locations (close to 90% female) might have implications to longnose dace health and long-term population viability at these sites because longnose dace have very small home ranges [24].

## 2. Methods

**Figure 1:**
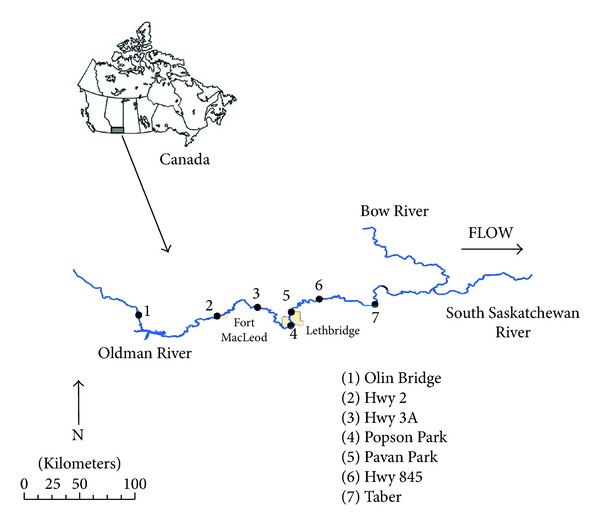
Map of Canada, the location of the Oldman River basin in southern Alberta and sites sampled (solid dots) for water and longnose dace (*Rhinichthys cataractae)* during August and September, 2009. Site names correspond to those used in subsequent figures.

The Oldman River originates in the Canadian Rocky Mountains and flows east across southern Alberta through Fort MacLeod, Lethbridge, and Taber (populations of 3.072, 74.637, and 6.280, resp. [25]). Wastewater, plus cattle and crop production are the largest potential sources of 28 organic contaminants that we assayed in Oldman River water. Lethbridge discharges tertiary-treated wastewater [23]. About one third of the watershed's land cover (28000 km^2^) is agricultural and contains intense livestock operations [26]. Water samples and longnose dace (*Rhinichthys cataractae*) were collected from the same sites as used by Jeffries et al. [10, 23], with exception of their most downstream site at Highway 36, (Figure 1) to facilitate comparison.

### 2.1. Study Species and Sampling

Longnose dace (*Rhinichthys cataractae*) are an abundant Cyprinid. Adults average about 6 cm in length and live to a maximum of 5 years [27]. Females are typically slightly larger and longer-lived than males. Longnose dace spawn multiple times from May to early August and is generally a benthic species that feed primarily on aquatic insect larva [27, 28]. Longnose dace are likely an important forage species for *Salmonids* and other larger fish and dace are a good candidate for a sentinel species approach because they have a high recapture rate and small home range [24].

### 2.2. Water Samples

 All glassware used in the collection and extraction of organic residues was silylated with dichlorodimethylsilane solution (Sylon CT) following the procedure of Ikonomou et al. [29]. Before each use all glassware was cleaned with acetone and baked at 325°C for four hours to remove any trace organics. Water samples were taken in early October 2009 during low flow to reflect base flow conditions (peak discharge typically occurs in June in the Oldman River). Three, 4 L amber glass bottles were prerinsed and filled with unfiltered river water from each site to make a total 12 L sample. The samples were stored on ice during transport and at 4°C upon returning to the laboratory until extraction, which was done within 24 hours of collection to minimize sample degradation. Twenty-eight organic contaminants, which can be divided into eight functional groups to determine potential sources (Table 1), were analyzed in all water samples. Three liters of river water were transferred to each of 4, 4 L Erlenmeyer flasks and the contaminants were extracted with constant stirring for 2 hours into 300 mL of dichloromethane (DCM) added to each flask. All river water samples were spiked with a mix of five labeled internal standards before extraction (with concentrations in ng mL^−1^ in square brackets (Ring-^13^C_6_-nonlyphenol [0.6], Propane-*d*_6_-bisphenol A [4.0], Di-*n*-octyl phthalate-*d*_4_ [2.0], b-Estradiol-17-acetate [3.0], and 2,2,3,4,4,6-*d*_6_-cholesterol [10.0]) to determine the extraction efficiencies. Following extraction the DCM phases were separated from the aqueous phases in a 2 L separatory funnel, then the 4 DCM samples were combined and concentrated by rotary evaporation to produce 10–12 mL of DCM phase from 12 L of river water. Procedural blanks using spectrophotometer grade water were also performed to check for contamination following the same procedure as the river water. Extracted water samples were sent on ice to the Institute of Ocean Sciences in Sydney, BC, Canada for further processing and quantification using gas chromatography-high-resolution mass-spectroscopy-based analytical procedures outlined in Ikonomou et al. [29].

### 2.3. Fish Samples

Longnose dace were sampled between mid-August and early-September 2009-using a-backpack electrofisher (model 12-B POW Smith-Root Inc.) to stun fish followed by collection with 6 mm mesh size dip nets. Electrofishing continued until at least 30 adult longnose dace at least 5 cm in fork length were caught. Based on previous experience, longnose dace that met this approximate length requirement usually had sexually developed gonads. If an individual fish did not have visible gonads then tissue was not used for further analysis regardless of fork length. If more than 30 dace were caught, then 30 were randomly selected to be sacrificed and if available, an additional 20 fish from each site were randomly selected, weighed, and measured (either adults or juveniles) for additional length-weight data. Fish were measured (fork length ±1 mm) and weighed (±0.01 g) to calculate condition factor (weight/fork length^3^  ×  10^6^). Fish were either sacrificed in the field with a quick cut of the spinal cord or returned to the laboratory in a cooler with a bubbler and sacrificed the next day. In the laboratory, the fish were anesthetized using ethyl 3-aminobenzoate methanesulfonate salt (MS-222) before they were euthanized. All adult dace were dissected to assign sex based on visual appearance of their gonads. The liver and gonads were weighed to calculate hepatosomatic (HSI) and gonadosomatic (GSI) indices (organ weight/(body weight—organ weight) × 100). The liver and one gonad was immediately frozen in liquid nitrogen and stored at −80°C for later real-time polymerase chain reaction (RT-PCR) analysis. The other gonad was stored in neutral buffered 10% formalin for 5–7 days, then transferred to 70% ethanol until later histological analysis to investigate signs of intersex gonads.

Juvenile dace from Popson Park were collected in late August, 2009 to serve as a reference group. Fish were taken from Popson Park because juveniles were abundant and a sufficient sample size of 26 was achieved for laboratory study. At collection these fish had a mean fork length of 2.99 cm and a mean weight of 0.34 g. The fish were kept in an 80 L tank from late August, 2009, to early March, 2010, to allow time for them to grow and sexually mature. Tank water was circulated through two filters equipped with activated carbon and every week 50% of the tank water was replaced with distilled water further purified by reverse osmosis. The fish were fed flake goldfish food (Aqueon) or bloodworms (Kyorin) once a day. All reference site fish were adults by the time they were sacrificed in early March and their liver and one gonad was removed for quantitative reverse transcription polymerase chain reaction (RT-PCR) analysis.

### 2.4. RNA Extraction, RT-PCR and Histology

Liver mRNA was extracted from 10 females and 10 males (if available) from each site and all reference fish and placed in TRIZOL Reagent after homogenizing with 18 and 21 G needles. The RT-PCR procedure used to quantify vitellogenin expression has been outlined previously [25] and described in detail elsewhere [30]. The only difference from Jeffries et al. [23] was that goldfish vitellogenin primers were used and Glyceraldehyde 3-phosphate dehydrogenase (GAPDH) was amplified for each vitellogenin sample as an internal control. Results are reported standardized to GAPDH. Due to the average size of longnose dace and the associated difficulty in obtaining blood samples, vitellogenin mRNA was used rather than blood plasma vitellogenin protein levels.

Gonads were prepared for histology following standard procedures. The dehydrating, clearing, and infiltrating was automated with an Leipca Model 1020 machine. Five males and four females from each site were randomly selected for this procedure. After the gonads were embedded in paraffin wax, 6 *μ*m sections were sliced using a microtome, mounted on microscope slides, and stained with hemotoxin and eosin dyes. The sections were examined under a light microscope at 100–400x magnification to confirm sex and to assess the presence of intersex. 

### 2.5. Statistical Analyses

Condition factor, GSI, HSI, and vitellogenin mRNA levels were tested for normality and homogeneity. Homogeneity of variances were checked using *F*-max tests and normality was checked using Shapiro-Wilk tests. Condition factor, GSI, and HSI were found to be normal and homogenous and analyzed with one-way Analysis of Variance (ANOVA) and Tukey's pairwise comparisons. Liver vitellogenin mRNA levels were found to be heterogeneous and nonnormal. We therefore used the nonparametric Kruskal-Wallis test without any data transformations and multiple pairwise comparisons were conducted using the Bonferroni adjustment method. Sex ratio differences from an expected 50 : 50 ratio at each site were analyzed with chi-square tests. All statistics were run separately for males and females except condition factor and sex ratios. Because there were no significant differences found between males and females for condition factor, males and females were combined for this analysis only. All statistics were performed with a critical *α* of 0.05. 

## 3. Results

### 3.1. Endocrine Disrupting Compounds

Our contaminant analyses indicate that many of the compounds detected in April 2006 [10] were detected again in September 2009 (Table 1) and were detected at numerous sites sampled along approximately 300 km of the Oldman River. Similarly, many assayed compounds not detected in 2006 were also not detected in 2009. Many of the detected compounds increase in concentration with distance downstream, particularly for compounds with multiple sources such as cholesterol and its derivatives, and phytosterols. Natural hormones, synthetic estrogens, and *α*-zearalanol display sporadic detections spatially over the two samplings and when detected, they were at low concentrations; estrone and 17 *β*-estradiol were the most detected of this group with concentrations typically higher at downstream sites. The concentrations measured in September, a period when discharge is typically near its lowest for the year, were usually higher than those measured in April [10], yet were present in many cases in the same relative proportions.

### 3.2. CPUE, Condition Factor, HSI, and GSI

**Figure 2:**
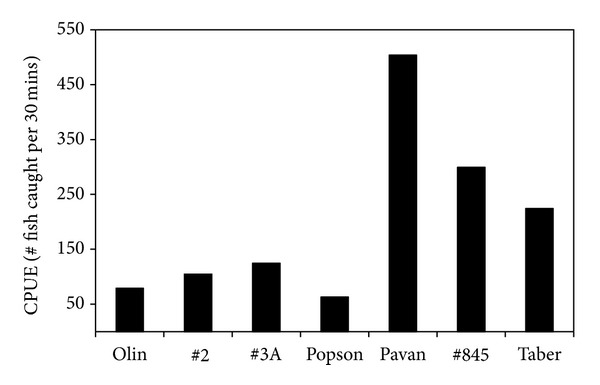
Catch per unit effort (number of fish per 30 minutes of electrofishing) of longnose dace (*Rhinichthys cataractae)* at each site on the Oldman River.

More fish were caught downstream of Lethbridge (Pavan Park, Highway 845 and Taber) than upstream (Olin bridge, Highway 2, Highway, 3A and Popson Park, Figure 2). Adult longnose dace captured downstream of Lethbridge (Pavan Park and Highway 845) had significantly higher condition factor then those captured upstream (e.g., Olin bridge and Highway 2) (ANOVA, *F* = 9.2858, *P* < 0.001), see Table 2. Adult females at the furthest upstream site (Olin Bridge) had significantly higher HSI then those at the site directly downstream of Lethbridge (Pavan Park, ANOVA, *F* = 3.264, *P* < 0.01), see Table 2. Males at the furthest upstream site (Olin Bridge) had significantly lower HSI then those downstream of Fort Macleod (Highway 3A, ANOVA, *F* = 3.6015, *P* < 0.01), see Table 2. Males at the closest upstream site from Fort MacLeod (Highway 2) and the furthest downstream site (Taber) had significantly higher relative gonad weights then males at the site directly downstream of Lethbridge (Pavan Park, ANOVA, *F* = 3.8554, *P* < 0.01), see Table 2. There were no significant differences in relative gonad weights between any of the sites for females (ANOVA, *F* = 1.715, *P* = 0.1216), see Table 2.

### 3.3. Sex Ratios and Gonad Histology

**Figure 3:**
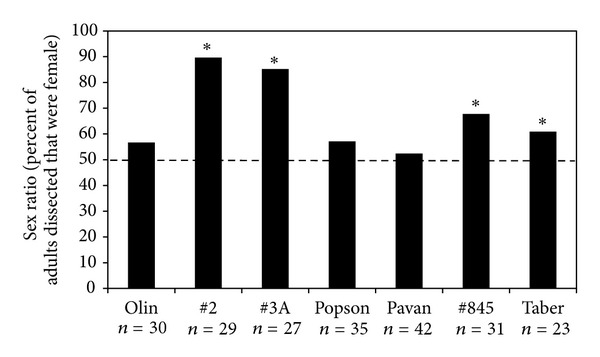
Sex ratios of longnose dace (*Rhinichthys cataractae*) collected from the Oldman River during August and September, 2009. Asterisks indicate that the sex ratio at the site is significantly different from 50 : 50 (dashed line for reference) at *P* < 0.05 as determined by chi-square analysis.

Longnose dace populations at two sites upstream of Lethbridge (Highway 2 and Highway 3A) were highly female-biased (85–89%) and significantly different from 50 : 50 (chi-squared test, *χ* ^2^ = 62.9,  *P* < 0.05,  *χ* ^2^ = 49.5,  *P* < 0.05, resp., Figure 3). Two sites located downstream of Lethbridge (Highway 845 and Taber), also had female-biased sex ratios significantly different then 50 : 50 but to a lesser extent (60–67%) (chi-squared test, *χ* ^2^ = 12.6,  *P* < 0.05,  *χ* ^2^ = 4.73,  *P* < 0.05, resp., Figure 3).

**Figure 4:**
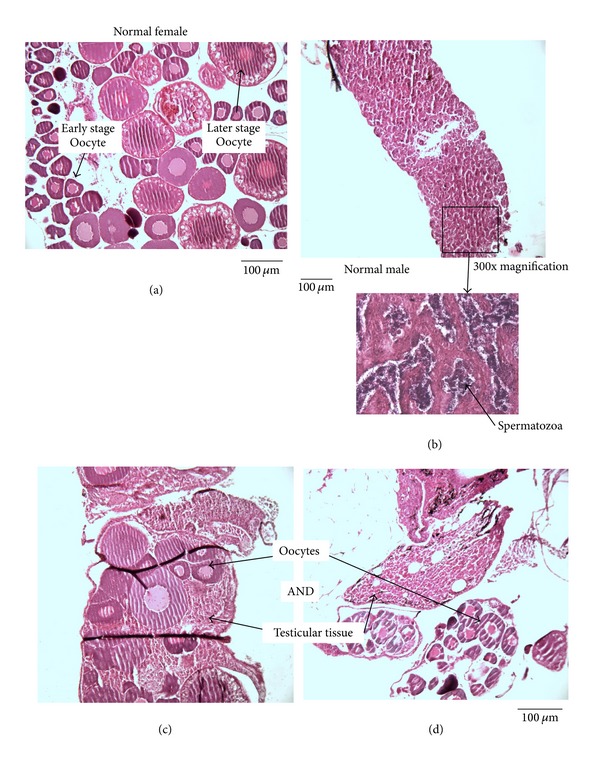
Stained sections of longnose dace gonads illustrating: (a) a normal female ovary showing different stages of oogenesis; (b) a normal male testes; (c) intersex gonads from Popson Park; (d) intersex testes from Highway 2. All fish were sampled from the Oldman River during August and September 2009. The dark lines in intersex gonads are wrinkles in the tissue produced by slicing. Scale bars are approximate.

Eleven percent of all adult longnose dace (male and female) assessed histologically contained intersex gonads; however, the occurrence of fish with intersex gonads was unevenly distributed among sites and was restricted to three sites upstream of Lethbridge (Highway 2, Highway 3A, and Popson Park) and one site downstream of Lethbridge (Taber). At these sites, up to 38% of fish contained intersex gonads. Interestingly, a number of gonads identified as intersex had similar amounts of ovarian and testicular tissue (Figure 4). A comparison of fish whose sex was determined histologically and by visual inspection of the gonads at dissection (excluding those fish with confirmed intersex gonads) revealed a 3% discrepancy between the two techniques (2 of 61 fish).

### 3.4. Liver Vitellogenin mRNA

**Figure 5:**
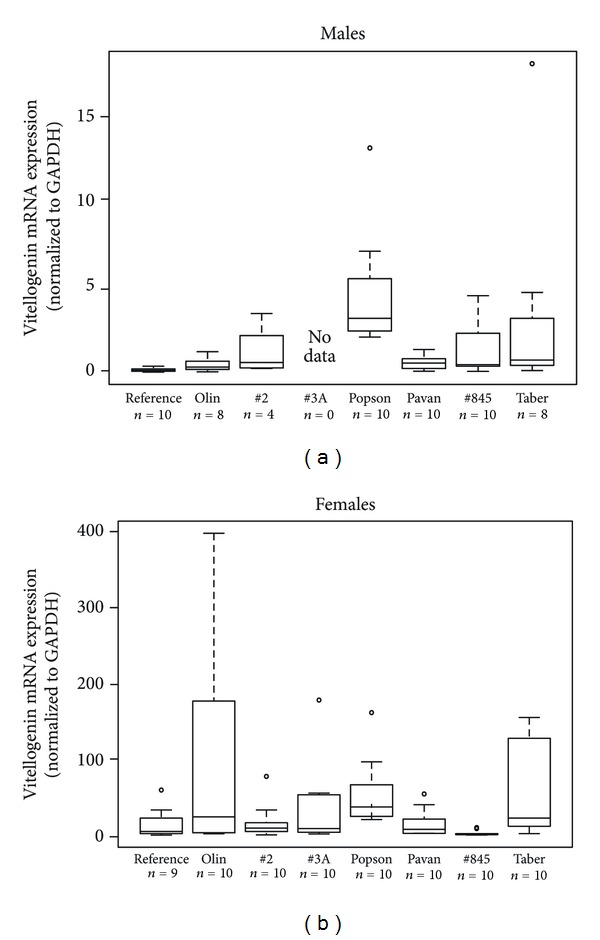
Box plots illustrating normalized liver vitellogenin mRNA expression in male (a) and female (b) longnose dace (*Rhinichthys cataractae*) from the Oldman River, Alberta sampled during August and September 2009. Vitellogenin mRNA expression is normalized to GAPDH. There were insufficient males collected from Highway 3A to analyze male vitellogenin mRNA. The box plot labeled “reference” is for dace collected at Popson Park and reared in the laboratory for 7 months in clean water and commercial food.

 At all sites female longnose dace had higher mean liver vitellogenin mRNA levels than males. Males at one site upstream of Lethbridge (Popson Park) had significantly higher liver vitellogenin mRNA levels then fish at other sites upstream and downstream of Lethbridge (Olin Bridge and Pavan Park), as well as the reference fish (Kruskal-Wallis test, *H* = 31.5297, *P* < 0.001) (Figure 5). The reference, laboratory-reared males had the lowest liver vitellogenin mRNA expression compared to males analyzed immediately after sampling. Fish at the furthest downstream site (Taber) also had relatively high liver vitellogenin mRNA levels but it was not significantly higher than any other site. Females at the most upstream site (Olin Bridge) had the highest liver vitellogenin mRNA levels and were significantly higher than other sites upstream and downstream of Lethbridge (Highway 2, Pavan Park, and Highway 845, Kruskal-Wallis test, *H* = 27.1711, *P* < 0.001, Figure 5).

## 4. Discussion

 Our results indicate that adult longnose dace sex ratios have remained (relative to 2005) heavily female-biased at many sites in the Oldman River and on average 11% of fish collected that were evaluated histologically had intersex testes. This provides strong evidence for the hypothesis that dace populations in the Oldman River have been feminized, presumably due to the presence of compounds with estrogen-like activity. Furthermore, the assignment of sex by visually inspecting gonads was found to be very accurate when contrasted to the histological analysis (97% congruence) thus providing confidence in the sex ratio assignment despite the lack of a known master sex determinant gene. Female-biased sex ratios occurred at sites upstream and downstream of Lethbridge where we also detected veterinary drugs, suggesting agricultural, nonpoint sources of EDCs. Highway 2 had the highest proportion of females and is upstream of Fort Macleod, the first municipal wastewater input. Longnose dace spawn multiple times throughout their breeding season [28], which also coincides with pesticide and manure application to fields [23]. Thus, longnose dace may be exposed to relatively high concentrations of compounds with estrogen-like activity during a critical time of their sexual development (i.e., before hatching, fry stage, etc.) as indicated by elevated vitellogenin mRNA levels in males. Estrogen-like compounds have been shown to bias sex ratios towards females in Japanese medaka (*Oryzias latipes*) laboratory exposures at an early stage of development [31].

Skewed sex ratios could also result from sampling error or sex-specific mortality. However, the sampling methodology was consistent between sites and we took care to sample similar river habitat at each site. We have employed the same sampling protocols in other adjacent, major southern Alberta rivers (Bow and Red Deer [10, 23, 32]) and not found evidence for such highly female-biased sex ratios. Male longnose dace commonly develop orange coloration during spawning [27],which may make them more conspicuous to predators at that time. However, the idea that only at certain sites males are selectively preyed upon because of predation pressures is not supported by our recent observations demonstrating significant male bias in longnose dace population in other rivers in Southern Alberta (unpublished data). 

Intersex fish were found at multiple locations in the Oldman River yet basin-wide instances of intersex gonads were not very common (11%) and are comparable to other studies on white suckers where 18–22% of fish were intersex downstream of Boulder, Colorado [18]. In gonochoristic species such as longnose dace, intersex gonads are not a normal condition, although it may be seen in some fish species with tendency for natural hermaphroditism [33]. This is supported by the observation that no intersex gonads were observed at the most upstream and relatively unimpacted reference site (Olin Bridge). Our observations of highly female-biased sex ratios, and increased expression of vitellogenin mRNA in males in areas with high incidence of intersex gonads are consistent with the hypothesis that exposure to estrogen-like endocrine disruptors resulted in abnormal gonadal development and feminization of longnose dace populations in the Oldman River. In this context, the sites where intersex fish were found correlates with the highest female-biased sex ratios and the presence of confirmed estrogen-like contaminants. Unfortunately, we cannot test this hypothesis further because there is no known sex-determinant gene marker in this species. 

Male longnose dace at Popson Park, which is upstream of Lethbridge, had significantly upregulated liver vitellogenin mRNA compared to the furthest upstream site (Olin bridge). Furthermore, laboratory-reared males had very low levels of liver vitellogenin mRNA following growth in clean water and on a consistent diet (seven months). This is diagnostic of exposure to estrogenic compounds in the field since vitellogenin is an egg yolk precursor protein that is normally present at higher concentrations in females under control of estrogen [12]. Unexpectedly, there was no significant upregulation of vitellogenin immediately downstream of Lethbridge, which can be explained, in part, by the potential presence of pharmaceuticals downstream of municipalities [34] that interfere with normal estrogen signaling. Another contributing factor could be small male sample sizes at the sites with the most extreme female-bias, which makes it more difficult to demonstrate statistically significant differences in male vitellogenin expression. It should be noted that wastewater effluent is not the only source of contaminants, and agricultural land use contributes to the mixture of compounds with estrogen-like activity. This is consistent with our results demonstrating the presence of natural and synthetic hormones such as *α*-zearalanol (a veterinary growth promoter used in many feedlots), which are present in abundance north of Lethbridge in subcatchments that drain into the Oldman River. Endocrine disruption of aquatic organisms has been documented in other areas with intense agricultural activity [7, 35]. A lack of correlation between female-biased sex ratios and higher levels of vitellogenin observed at certain locations may result from exposure of compounds with androgenic, antiandrogenic or antiestrogenic activities, which may interfere with estrogen-induced vitellogen expression. Bing et al. [36] found the pesticide pentachlorophenol to be antiestrogenic and to reduce vitellogenin production in juvenile goldfish. It is also possible that some compounds may interfere with the production of vitellogenin by binding to, and inhibiting, the estrogen receptors [37–39]. Thus, complex mixtures of compounds with different types of activity likely exist in the Oldman River and can simultaneously act on the endocrine system of male and female dace in different ways. 

Our findings were consistent with Jeffries et al. [23] as some sites downstream of the most upstream site (Olin Bridge) had female-biased sex ratios and elevated vitellogenin mRNA levels in males. Differences in our results and those of Jeffries et al. [23] are likely due to the differences in sampling year and season. For example, Jeffries et al. [23] found significant female-biased sex ratios at different sites than did we. Jeffries et al. [23] sampled in April 2005 and it is not surprising that yearly and seasonal differences in river flows and runoff could lead to different concentrations of EDCs. Vitellogenin mRNA levels might also be different since we sampled postreproduction in late summer and fall as opposed to prereproduction in the spring. We note that vitellogenin mRNA expression cannot be directly compared because different housekeeping genes were used during the RT-PCR. However, both studies demonstrate spatial and temporal consistency for evidence of endocrine disruption in longnose dace in the Oldman River. 

 We found, similar to Sosiak and Hebben [34] and Jeffries et al. [10], that many of the 28 compounds we attempted to measure can be detected over large spatial scales in the Oldman River, a result that confirms not only the consistency of the presence of many of these compounds, but also their widespread spatial occurrence in the Oldman River basin. Our snapshot in time was not designed to reconstruct the exposure history of contaminants experienced by longnose dace in the Oldman River because contaminant concentrations will change as a function of river discharge, and additional compounds exist [34] that we did not attempt to quantify. While the behavior of many of these compounds is not well known, many are relatively soluble and, therefore, have the potential to be taken up from the water by biota as a result. Nevertheless, the two samplings, which cover different times of the year and the hydrologic cycle, indicate that there is consistency in the detection of many of these compounds over different years and seasons. Variation in concentrations would be expected due to short-term weather that affects the movement of compounds from surrounding landscapes to the river and dilution of the relatively constant wastewater treatment plant effluents. Our data provide an indication of the variety of compounds to which Oldman River biota are exposed, and the large spatial extent of many of the detected residues. 

 Extreme cases of endocrine disruption in fish may lead to population collapse [40]. In a lake in the Experimental Lakes Area (ELA) of Northern Ontario fathead minnows were exposed to chronic, low levels of synthetic estrogen added for three years [40]. At the experiment's conclusion, males were feminized, females exhibited delayed oogenesis, which likely affected their ability to reproduce, and, ultimately, a near extinction of the fish in the lake occurred [40]. Evidence suggests that EDCs can impact fish reproductive success [41] and delayed oogenesis can be detrimental to female reproductive success if females do not have sufficient time to breed or if fry experience insufficient growth prior to winter. Males also show signs of hindered reproductive success by having smaller testes [7, 42] and reduced sperm motility [43]. Smaller gonads and reduced egg production has also been found in female fish exposed to EDCs [42, 44]. We do not know the implications of the severely female-biased sex ratios that we have seen to long-term dace population viability in the Oldman River. We also do not know if the few males without intersex testes or upregulated vitellogenin present at these sites possess adaptive genetics that allow them to persist while others do not. We are in the process of identifying potential adaptive genes whose regulation could be measured at these and other sites. For example, metallothionines are produced in response to exposure to trace metals, and they bind to excess metals that would, otherwise, lead to cellular metabolic toxicity. There are likely protective analogues for metals in animals and possibly other classes of compounds like EDCs and if so, there may be an adaptive advantage to fish that could produce such compounds. There may also be genetic adaptations by fish that live in contaminated sites; for example, a six-base deletion in aryl hydrocarbon receptor 2 (AHR2) has recently been identified in Hudson River Atlantic tomcod (*Microgadus tomcod*) that impairs the ability of the receptor to bind TCDD or PCB126 [45], thereby effectively reducing toxicity.

Elevated nutrient inputs are likely responsible for longnose dace having higher condition factor and higher abundance at sites downstream of Lethbridge. Water quality generally decreases with distance downstream in the Oldman River with both higher nitrate and total phosphorus levels [23]. Municipal wastewater effluent and nonpoint sources such as basin-wide agricultural activity contribute to the change in water quality. Therefore, it makes sense that increased productivity generates a higher abundance and heavier individuals. This trend was also found in longnose dace previously in the Oldman, Bow, and Red Deer Rivers [23, 32] It is generally accepted that nutrient enrichment of rivers leads to greater fish productivity [46] However, fish that appear to be healthy when they are exposed to waste water effluent may be physiologically stressed. 

There was not a clear pattern for relative gonad and liver weights (HSI and GSI). Increased liver size in fish is related to food intake and energy storage [47], which means that fish at downstream sites should have larger liver weights. This was, however, not the case in this study. Increased food availability also allows fish to invest more into their gonads. Conversely, compounds with estrogen-like activity have been shown to reduce gonad size in fish in the laboratory [48]. However, male and female fish did not consistently have smaller gonads at downstream sites. There may have been opposing effects of increased nutrients/food availability and exposure to endocrine disruptors in the river. The lack of a clear pattern, particularly for gonad mass, may also be because we sampled longnose dace after their spawning season [27]. Liver and gonad weights in fish are sensitive to different phases of the reproductive cycle [47]. Differences would be more obvious just before or during spawning since there is more energy investment in the liver and gonads. Longnose dace had higher HSI and GSI downstream of Lethbridge in an earlier study [23] and this discrepancy may reflect an early April sampling, prior to longnose dace spawning.

## Figures and Tables

**Table 1:** Concentrations are ng/12 L of 28 target compounds measured by ultratrace analytical gas chromatography-high-resolution mass-spectrometry in river water from 7 sites on the Oldman River, Alberta, Canada during September, 2009. Many of these compounds are hormonally active or are suspected to be hormonally active. The contaminants are grouped according to their functionality (headings in italics) to facilitate identification of potential sources and are used in subsequent interpretations.

Target compound	Olin bridge	Highway 2	Highway 3A	Popson	Pavan	# 845	Taber	Description or use
*Natural h* *ormones*								
Estrone	ND^a^	ND	ND	0.15	0.15	0.72	0.74	Endogenous estrogen
17a-Estradiol	ND	ND	ND	ND	ND	ND	ND	Endogenous estrogen
17b-Estradiol	ND	ND	0.54	0.20	ND	0.35	ND	Endogenous estrogen
Estriol	ND	ND	ND	ND	ND	ND	ND	Endogenous estrogen
Testosterone	1.03	ND	ND	ND	ND	0.42	ND	Endogenous androgen
*Synthetic e* *strogens*								
Equilin	ND	ND	ND	ND	ND	ND	ND	Hormone replacement therapy
d-Equilenin	ND	ND	0.20	ND	ND	ND	ND	Hormone replacement therapy
Mestranol	ND	ND	ND	ND	ND	ND	ND	Synthetic ovulation inhibitor
19-Norethindrone	ND	0.42	ND	ND	0.49	ND	0.42	Synthetic ovulation inhibitor
17a-Ethynylestradiol	ND	ND	ND	ND	ND	ND	ND	Synthetic ovulation inhibitor
(−)-Norgestrel	ND	ND	ND	ND	ND	ND	ND	Synthetic ovulation inhibitor
*Veterinary pharmaceuticals*								
a-Zearalanol	ND	ND	ND	ND	ND	ND	6.16	Growth promoter
b-Estradiol-3-benzoate	ND	ND	ND	ND	ND	ND	ND	Growth promoter
*Industrial compounds*								
Bisphenol A	10.75	ND	2.25	2.08	13.67	12.33	11.67	Plastics catalyst
*Cholesterol and derivatives*								
Cholesterol	191.17	162.22	314.92	339.33	444.50	551.67	387.83	Animal/plant sterol
Desmosterol	121.67	198.25	ND	399.75	ND	50.57	66.03	Cholesterol derivative
Cholestanol	31.17	51.08	119.83	121.92	229.33	29.77	222.92	Cholesterol derivative
6-Ketocholestanol	ND	ND	ND	ND	ND	ND	0.70	Cholesterol oxidation product
7-Ketocholestanol	4.86	ND	ND	ND	ND	12.99	13.44	Cholesterol oxidation product
*Coprostan-3-one*	6.53	ND	ND	ND	ND	106.95	141.88	Fecal neutral sterol
*Ergosterol*	17.18	ND	ND	ND	ND	33.97	28.58	Sterol produced by fungi
*Phytosterols*								
b-Sitosterol	145.50	111.50	154.5	186.25	257.33	296.00	233.25	Phytosterol
Campesterol	14.08	18.17	23.17	25.25	33.00	40.67	30.41	Phytosterol
Stigmastanol	41.42	20.33	157.25	62.17	124.25	170.20	119.58	Phytosterol
Stigmasterol	46.75	88.92	101.67	99.50	119.67	148.58	13.21	Phytosterol
Fucosterol	134.33	106.67	141.92	163.48	190.92	228.25	185.25	Seaweed Sterol
Totarol	ND	ND	ND	ND	ND	ND	ND	Antibacterial diterpenoid
Pinosylvin	ND	ND	ND	ND	ND	ND	ND	Stilbene found in *Pinus* sp.

^a^ND: not detected.

**Table 2:** Condition factor for all adults together and HSI and GSI for male and female longnose dace (*Rhinichthys cataractae*) collected in the Oldman River (AB, Canada) during August-September 2009^a^.

Site	Condition factor (All adults)	Female HSI	Female GSI	Male HSI	Male GSI
Olin bridge	8.957 ± 0.140 A	2.011 ± 0.157 A	2.029 ± 0.194 A	1.006 ± 0.123 A	0.701 ± 0.0590 ABCD
Highway 2	8.607 ± 0.137 A	1.427 ± 0.144 AB	2.305 ± 0.168 A	1.290 ± 0.257 AB	1.167 ± 0.294 ACD
Highway 3A	8.889 ± 0.175 A	1.636 ± 0.132 AB	2.463 ± 0.152 A	2.201 ± 0.734 B	0.723 ± 0.0556 ABCD
Popson park	8.789 ± 0.119 A	1.392 ± 0.0994 AB	1.896 ± 0.182 A	1.140 ± 0.109 AB	0.767 ± 0.0593 ABCD
Pavan park	9.521 ± 0.145 B	1.255 ± 0.177 B	2.020 ± 0.0378 A	1.223 ± 0.121 AB	0.609 ± 0.0367 B
Highway 845	9.553 ± 0.0879 B	1.839 ± 0.190 AB	1.886 ± 0.177 A	1.804 ± 0.209 AB	0.617 ± 0.0381 ABC
Taber	8.836 ± 0.140 A	1.583 ± 0.159 AB	1.891 ± 0.248 A	1.786 ± 0.351 AB	1.103 ± 0.273 D

^a^Capital letters that are different within columns indicate statistically significant differences.
